# The demographic impact on the demand for emergency medical services in the urban and rural regions of Bavaria, 2012–2032

**DOI:** 10.1007/s10389-015-0675-6

**Published:** 2015-05-26

**Authors:** Alexander Veser, Florian Sieber, Stefan Groß, Stephan Prückner

**Affiliations:** Institute for Emergency Medicine and Management in Medicine - INM, Klinikum der Universität München, Schillerstr. 53, 80336 Munich, Germany

**Keywords:** Demand trends, Demography, Emergency medical services, Regional health planning, Socioeconomic factors, Urban–rural differences

## Abstract

**Aim:**

In most regions of the world, the proportion of older people in the population has increased during the last decades. As this entails major consequences for the healthcare sector, this study isolates and quantifies the impact of an aging population on the demand for emergency medical services in different types of regions in Bavaria between 2012 and 2032.

**Methods:**

Dispatch data of the emergency medical services were combined with population data and forecasts. Age-specific rates of emergency ambulance dispatches were calculated and used for a 20-year-projection for all 71 rural and 25 urban districts of Bavaria. Tests for differences between these two types of regions were applied.

**Results:**

Per capita rates of emergency ambulance dispatches in urban regions tend to be higher and there is an urban–rural distinction in the rates of specific age groups. The projection predicted an overall increase in emergency ambulance dispatches by 21 % in Bavaria within 20 years, solely due to demographic effects. At the regional level, this demographic impact ranged from about −3 % to +41 %. There is a clear urban–rural distinction and the 28 regions with the strongest increase are all rural regions.

**Conclusion:**

The substantial demographic impact in combination with strong urban–rural variations should be accounted for in regional long-term planning as well as age-group specific innovation in the emergency medical services. As demography is not the only significant demand factor, the identification and quantification of other factors remains a challenge for further research.

## Introduction

In most regions of the world, the proportion of older people in the population has increased during the last decades as life expectancy has gone up and the number of births has gone down. This aging process is expected to continue across the world and is especially advanced in developed countries like those of the European Union or Japan (European Commission 2013; United Nations et al. 2013a, b).

Aging has major consequences for population health and the healthcare sector. Higher age entails a higher occurrence of chronic diseases and multi-morbidity (Barnett et al. 2012; Nowossadeck 2012; Prückner and Madler 2009). As a consequence, old people are hospitalized more frequently and for longer periods than young people (DESTATIS 2010). Aging has accordingly been identified as an important contributor—amongst others such as advancing medical technology—to rising health costs (Breyer et al. 2010).

Emergency medical services (EMS) are equally affected by aging. Old people are markedly more often patients of EMS than young people (Behrendt and Runggaldier 2009; Lowthian et al. 2011a; McConnel and Wilson 1998; Prückner et al. 2008). Several studies have documented a fast rising demand for EMS over the past decades in developed countries like Australia (Lowthian et al. 2011a), Germany (Schmiedel and Behrendt 2011), Japan (Hagihara et al. 2013; Sasaki et al. 2010), Spain (Díaz-Hierro et al. 2012), the UK (Peacock et al. 2005; Wrigley et al. 2002) or the USA (Burt et al. 2006). Some authors have linked at least some of this rise to demographic changes and have provided projections of the total future demand for EMS in their respective study area, considering demography either as sole (Behrendt and Runggaldier 2009; Hagihara et al. 2013; Lowthian et al. 2011a) or as one of two (Lai and Wong 2012; Sasaki et al. 2010) predictor variables.

This study isolates and quantifies the impact of demographic changes on the demand for EMS in the German Federal State of Bavaria in a regional perspective. As our data allows a comparison of all 96 Bavarian regions, we can reveal striking urban–rural differences in the age-specific demand for EMS in 2012 and its projected development in the future. Our results will inform decision-making about future regional supply structures in aging societies.

## Methods

### Study area

Bavaria is the largest of the 16 German federal states and comprises metropolitan areas such as Munich as well as peripheral regions such as the Bavarian Forest bordering the Czech Republic. For means of administration, Bavaria is subdivided into 96 districts, which are further categorized in districts made up of only one urban municipality—a major town or a city—and districts made up of several municipalities—towns and villages in predominantly rural areas. Relating to this official categorization based on settlement structure, we refer to the Bavarian districts as either rural regions (71 districts, officially *Landkreise*) or urban regions (25 districts, officially *kreisfreie Städte*). In 2012, the median population density in the urban regions was 1.036 inhabitants per square kilometer and 121 inhabitants per square kilometer in the rural regions.

According to official projections, Bavaria’s population size of approximately 12.5 million people in 2012 will slightly increase by 2.8 % within the next 20 years; however, demographic changes will have a huge impact on the age structure. By 2032, the number of inhabitants aged 65 years and older will increase by almost 40 %, whereas the population in the younger age groups will decrease by about 6 %. These trends, however, will take a regionally very diverse shape with increasing, stable and decreasing total populations and a varying intensity of the aging process (LfStaD 2014).

### Database

Our calculations of EMS per-capita demand in the year 2012 were based on official population data by the Bavarian state office for statistics as well as two de-identified EMS data sources. One source of EMS data was the documentation of all dispatches by the 26 regional dispatch centers in Bavaria, including time and place of the associated EMS missions. Only emergency dispatches of ambulances designated for patient transport were considered in our calculations. The other source of EMS data was the documented patients’ age for all billed ambulance dispatches by the central billing agency for EMS in Bavaria.

Linked together, these data yielded patient age information for all billed emergency ambulance dispatches (EAD) in 2012, amounting to 0.65 million EAD, representing 74 % of all EAD in 2012. For the remaining 26 %, no billing data and, thus, patient age existed, in most cases, because there was no patient transport, which happens, for example, when several ambulances are dispatched to the same patient or when a patient rejects transport. To include these EAD in our calculations, we had to assume an age distribution correspondent to that of the billed EAD; the analysis of dataset properties which were available for both billed and unbilled EAD (e. g., reason for dispatch, time of day or seasonal distribution) did not indicate an age bias resulting from this approach.

The aforementioned data, including age information from billing data, were identically available for the year 2011, but not for other years. In order to ensure that our base year 2012 is not an outlier year and to check for substantial variations in the age-specific demand for emergency services between individual years, we used the 2011 data for a validity assessment of our calculation results based on 2012 data. The relative importance of demography as a factor for the demand for EMS was assessed combining official population data with dispatch center data for the complete period from 2004 to 2012. The projection of the impact of demographic changes on EMS was based on official historic population data and the official population forecast.

### Calculations

Rates of emergency ambulance dispatches per 1.000 inhabitants (READ) in 2012 were calculated individually for each Bavarian region and age group as thus:

$$ REA{D}_{patient\  age\  group}=\frac{dispatches\ .\  bille{d}_{patient\  age\  group}}{inhibitant{s}_{patient\  age\  group}}\times \frac{dispatches\ .\  documente{d}_{total}}{dispatches\ .\  bille{d}_{total}}\times 1000 $$

The effect of changes in the population on the demand for EAD in a prospective year was then calculated individually for each region as shown in the following. The projection outcome was, thus, the percentage change in the number of EAD solely due to demographic changes—the demographic impact.

$$ demographic\  impac{t}_{total}^{2032}=\left({\displaystyle \sum_{patient\  age}\frac{dispatches\ .\  bille{d}_{patient\  age}^{2012}}{dispatches\ .\  bille{d}_{total}^{2012}}\times \frac{inhibitant{s}_{patient\  age}^{2032}}{inhibitant{s}_{patient\  age}^{2012}}}\right)-1 $$

All combinations of the age-specific READ in the base year 2012 with the projected population data for the years 2013–2032 as well as with historic population data for the years 2004–2011 were calculated. Our calculation therefore yielded not only a forward projection, but also a backward projection for means of comparison with the actually documented number of EAD in the past. All database calculations were done in an Oracle Database 11gR2 environment, whereas all statistical analysis was done with IBM SPSS Statistics version 20.

## Results

### Patient age and per capita rates in Bavaria

In Bavaria as a whole, people aged 75 years and older accounted for 33 % of all emergency ambulance dispatches (EAD) in 2012. At that time, this group constituted only about 9 % of the total population. Persons aged 90 years and older were 7.9 times more often patients of EAD than the population average.

**Fig. 1:**
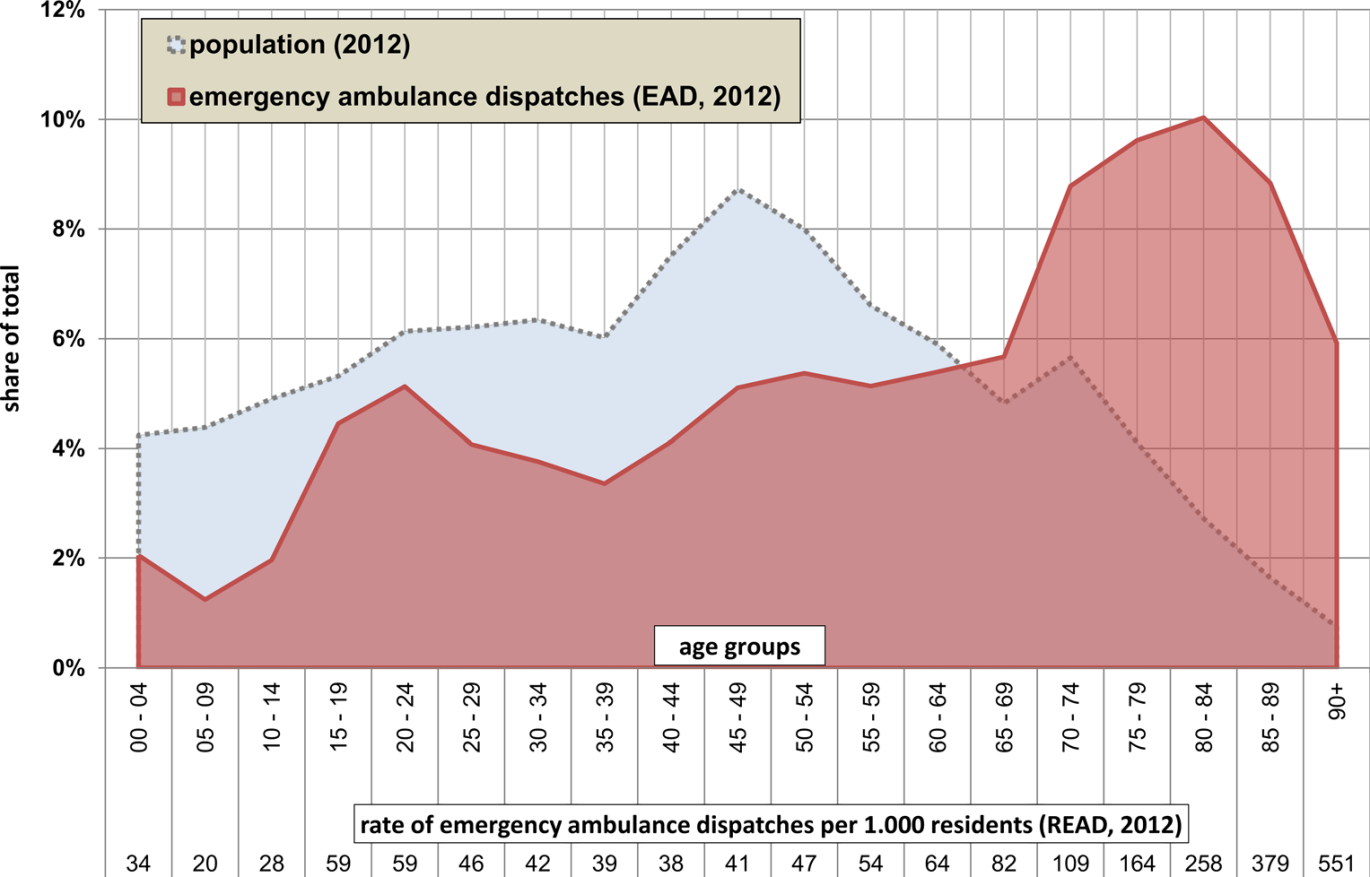
Emergency ambulance dispatches in Bavaria by age groups (2012)

Accordingly, the rate of emergency ambulance dispatches per 1,000 inhabitants (READ), which was 70 in total for all ages, rises with increasing age. This starts at about 40 years of age and becomes exponential from about 70 years of age on (Fig. 1). Amongst the younger age groups, children between 0 and 4 as well as youth and young adults between 15 and 24 years exhibit slight peaks in READ.

The comparison of the total READ for all ages in 2012 with the period 2004–2011 showed a steady increase from 50 in 2004 to 70 in 2012 at the aggregate Bavarian level. For the years 2011 and 2012, for which age-specific data was available, this upward trend was also true for the READ of most individual age groups.

The relations of the READ of one age group to the READ of other age groups, that is the relative probability for the individual age groups to become a patient of an EAD, were roughly the same in 2011 and 2012. A person aged 90+ was about 9.1 times more likely to become a patient of an EAD than a person aged 20–24 in 2011 and 9.4 times more likely in 2012.

### Regional per capita rates

**Fig. 2:**
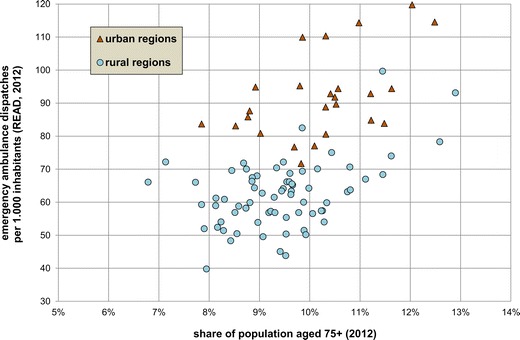
Rates of emergency ambulance dispatches per 1,000 inhabitants (READ) and share of population aged 75+ in the Bavarian regions (2012)

At the regional level, there is a large variation of the total READ for all ages, ranging from 40 to 120 within the 96 assessed regions in 2012 (Fig. 2). There is a clear distinction between rural and urban regions [unpaired *t*-test t(94) = 11.73, *p* < .001, READ mean difference of 29.6 (95 % CI, 24.6–34.6)]. All 62 regions with a READ below 72 are rural regions. Amongst the 21 regions with a READ above 83, only two are rural regions. Regions with a high share of population aged 75 years and older tend to also have a high READ, regardless of their settlement structure—Pearson’s *r*(94) = 0.516, *p* < .001.

The relations of the READ of one age group to the READ of other age groups vary widely between the regions. Depending on the region, the READ of age group 90 + years was between 4.9 and 16.0 times higher than that of age group 20–24 years. Clear distinctions of this relative READ were found between rural and urban regions for 13 of the 19 age groups [unpaired *t*-test respectively Welch’s *t*-test t(30–94) = 3.41–7.62, where *p* < .001]. Generally speaking, relative READ of several age groups below age 55 was distinctly higher in urban regions, whereas relative READ of several age groups above age 64 was distinctly higher in rural regions.

### Demography amongst other demand factors

Regression analysis confirms the strong connection between demography and the demand for EMS demonstrated in the previous sections. For use as predictor variable, we computed a standardized population aging index for each year and region based on the ratio of inhabitants 70 years and older over inhabitants below age 70. This aging index significantly predicts READ—*b* = 0.611, *t*(862) = 22.7, *p* < .001—and explains a significant proportion of READ variance—*R*^2^ = 0.372, *F*(1, 862) = 513.1, *p* < .001—for the period from 2004 to 2012.

Research suggests that, besides demography, the demand for EMS is also defined by a multitude of other factors (Lowthian et al. 2011b; Toloo et al. 2011). The composite effect of these other factors can be evaluated by the comparison of a backward projection of the *isolated* demographic impact on EMS with the number of actually documented *total* EMS dispatches in the past.

Official population data shows that between 2004 and 2011, a substantial aging process of the population had already been taking place in Bavaria. A backward projection based on this historic population data and our calculated per-capita rates for the year 2012 yields an average annual increase in EAD of 0.9 % solely through demographic changes between 2004 and 2011. During the same period, documented total EAD increased by an average annual 4.3 %. Consequently, an average annual increase of 3.4 % between 2004 and 2012 can be ascribed to factors which have exacerbated the demand for EMS regardless of demography and equally throughout the population.

### Projection of the regional demographic impact

We have shown in the preceding that demographic changes should have accounted for a yearly increase of 0.9 % in EAD between 2004 and 2012 at the aggregate level of Bavaria. In the 20 years between 2012 and 2032, the total population in Bavaria will increase by 2.8 % and the share of people aged 75+ will increase from 9.2 % to 12.7 %. For this period, we project an increase in EAD of 20.9 % solely due to demographic changes. This corresponds to an average annual increase of 1.0 % which will, however, not occur uniformly, but will be steadily declining from 1.4 % (2012–2013) to 0.6 % (2031–2032).

**Fig. 3:**
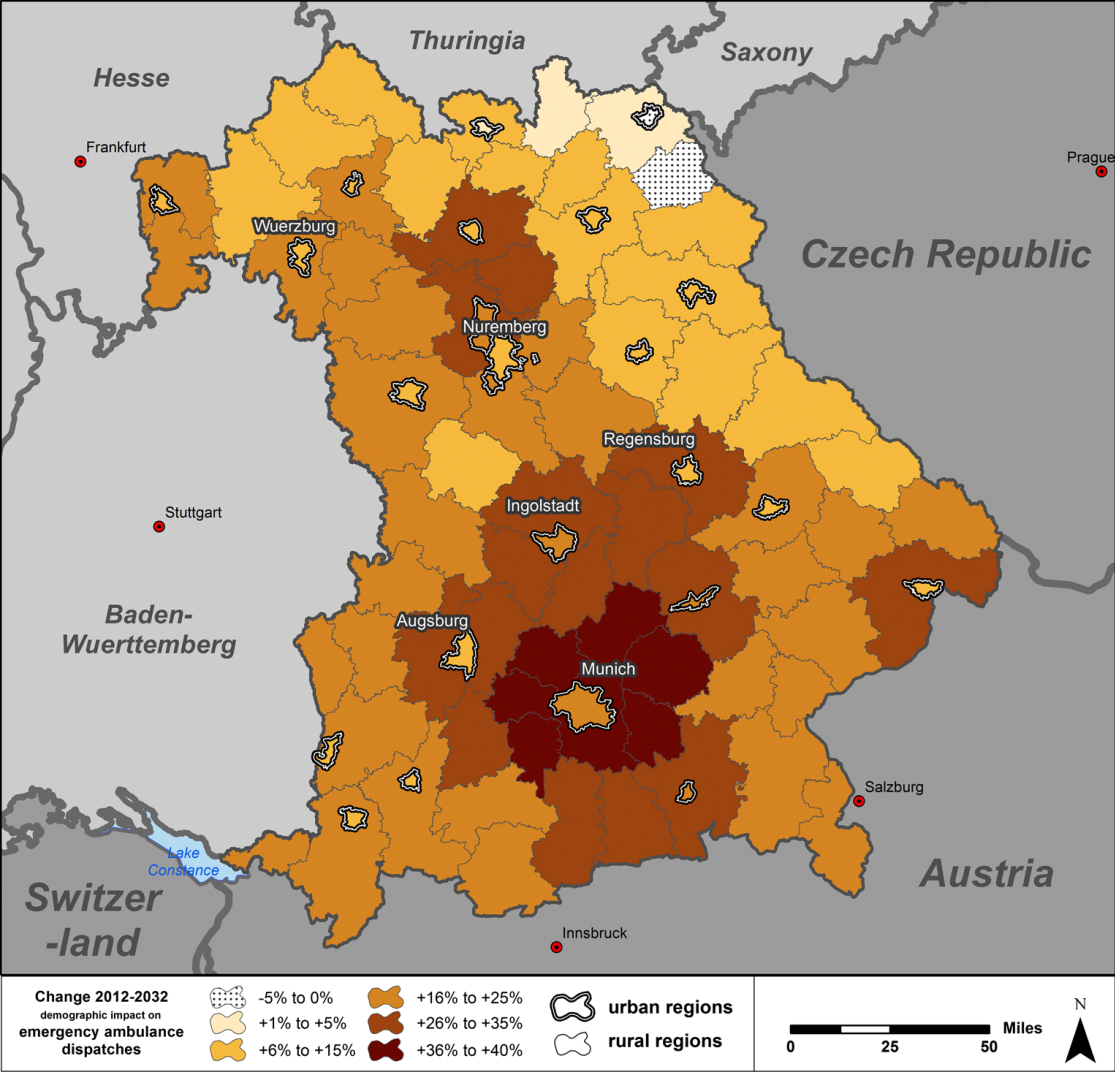
Projection of the demographic impact in urban and rural regions in Bavaria (2012–2032)

At the regional level, demographic changes are projected to contribute to a rising number of EAD in 94 of the 96 Bavarian regions between 2012 and 2032 (Fig. 3); thus, in spite of a decreasing total population in 45 regions, the aging process alone suffices to push up the number of EAD in all but two regions. With the mean at a 19.4 % increase and the median at an 18.9 % increase, the calculated demographic impact on EAD ranges from –2.8 % to +39.7 %.

There is a clear distinction between rural and urban regions [unpaired *t*-test *t*(94) = 3.98, *p* < .001, demographic impact on EAD mean difference of 8.1 % (95 % CI, 4.0–12.1 %)] and the 28 regions with the strongest increase are all rural regions. There is also a pattern in the relations between urban regions and their surrounding rural regions. In all but one of the 25 urban regions, the demographic impact is higher than in the respective surrounding rural regions.

## Discussion

### Validity

Our results confirm that age and emergency incidence are closely related, and that aging is an important driver of the demand for EMS. The juxtaposition of our calculation results at the aggregate level of Bavaria using data for our regular base year 2012 showed only minor deviations to identical calculations using 2011 as reference year. Other studies in Germany have also found age-specific rates of EMS (Behrendt and Runggaldier 2009; Prückner et al. 2008) similar to ours.

At the regional level, there are cases of substantial deviations between our calculation results using either 2011 or 2012 as base years in some regions and age groups. This can be traced back to small collectives forming some regional age groups, giving random events a high influence on the observed number of EAD in the individual years. In the region and age group with the greatest variation of EAD, the calculation of READ and demographic impact projections were based on the patient age information of 63 EAD in 2011 and 113 EAD in 2012.

As described in the ‘Results’ section, there must have been factors other than demography contributing to the increase in the demand for EMS in the past. In sum, these factors had a greater impact on demand than demographic changes. Other studies have also found that observed increases in the demand for EMS in the past could not solely be explained by demographic changes (Lowthian et al. 2011a; Sasaki et al. 2010). Accordingly, we perceive any projection of the *total* future demand for EMS based exclusively on demographic data as misleading. Hence, we explicitly restrict the validity of our calculations to the *isolated* percentage impact of demography on a total demand defined by many factors.

**Fig. 4:**
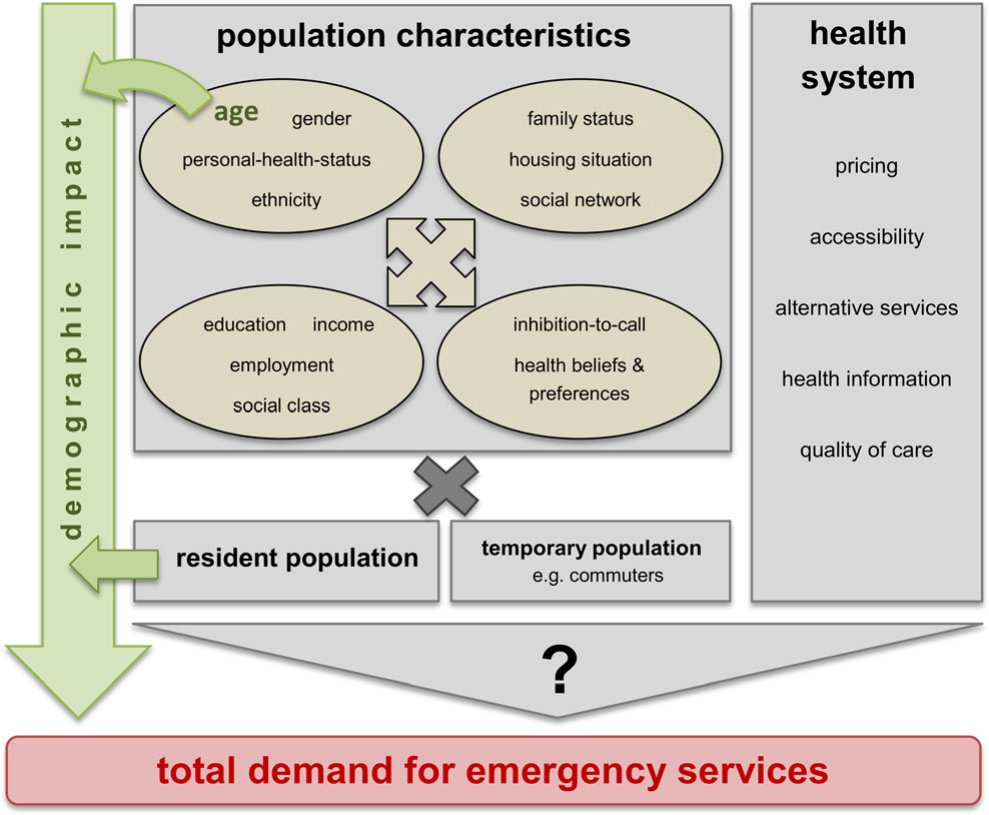
Factors for the total demand for emergency medical services. synopsis based on: David et al. 2013; Lowthian et al. 2011b; Siegrist 2009; Toloo et al. 2011, 2013; Wrigley et al. 2002

The following Fig. 4 gives an overview of such potential factors for the demand for EMS, with no claim to completeness, based on related literature and our own research results. These factors can basically be categorized into population characteristics like family status, education or health beliefs and preferences on the one hand and characteristics of the health system like pricing or alternative services on the other hand. In conjunction with the resident population size and the temporary population size, these factors determine the total demand for EMS. The operationalization of these factors and the investigation of their complex interactions, however, go far beyond the scope of this study focusing on the demographic impact.

### Strengths

To our knowledge, this is the first study to address the issue of demography and EMS demand in a regional differentiation. Similar studies have looked at single cities or metropolitan areas: Melbourne (Lowthian et al. 2011a), Hong Kong (Lai and Wong 2012), Niigata (Sasaki et al. 2010); or countries as a whole: Germany (Behrendt and Runggaldier 2009), Japan (Hagihara et al. 2013). The comparison of 96 regions with an identical legal framework and a rather homogenous culture has revealed drastic regional variations in the demand for EMS. We have found that urban regions tend to have a higher per capita demand for EMS than rural regions, that there are distinctions of the demand of individual age-groups between urban and rural areas, and that the impact of demographic changes in rural regions tends to be higher than in urban regions. There is not only a difference in demographic impact between urban and rural areas on average, but also in the particular relationship between urban regions and their respective surrounding rural regions.

The distinction in the scale of the demographic impact between urban and rural regions can be linked to distinctions in the degree of the future ageing process. Whereas in 2012, there was a difference of only about 1 % in the share of inhabitants aged 75+ between urban regions (median 10.3 %) and rural regions (median 9.4 %), the projected increase of inhabitants at age 75+ is distinctly higher in rural regions compared to urban regions [unpaired *t*-test *t*(94) = 5.21, *p* < .001, increase of inhabitants at age 75+ mean difference of 14.4 % (95 % CI, 8.9–19.9 %)]. With respect to the per capita demand distinctions between urban and rural regions, we suspect that the inflow of commuters into the urban regions contributes substantially to a higher emergency incidence there. This hypothesis is supported by the observed higher relative READ for age groups at school age and at working age in urban regions compared to rural regions. Thus, besides the total resident population, the temporary population of a region should also be considered as a factor for the total demand for EMS (Fig. 4).

### Limitations

In our projections, we assume that the legal and structural framework for EMS will not change and that, thus, help request and dispatching processes will remain similar as they are today. The population projections on which we base our calculations assume that trends in births and migration of recent years will continue in a similar form in the future (LfStaD 2014). Unlike other studies (Behrendt and Runggaldier 2009; Lai and Wong 2012; Lowthian et al. 2011a), we could not account for gender-specific emergency incidence rates, because we are lacking patient gender information.

The EMS data available to us did not allow for an evaluation of the long-term stability of the relative probability of people in different age groups to become a patient of EMS. Based on the compression of morbidity-hypothesis, it could be argued that increasing life expectancy in the future will shift high morbidity rates into ever older age groups. Contrastingly, the expansion of morbidity-hypothesis states that future gains in anticipated average life will be accompanied by added life years with high morbidity. To date, research on trends in morbidity has rendered ambiguous results. Depending inter alia on the health indicators and subpopulations examined, there is empirical evidence supporting compression, expansion as well as equilibrium of morbidity (Chatterji et al. 2014; Geyer 2014).

## Conclusion

We have shown that demography is one of the key factors for EMS demand. Its impact can be quantified as research has shown robust causalities between age and emergency incidence. At the same time, there is little that actors in the EMS system can do to change the course of demographic developments. This makes regionalized projections of the demographic impact especially valuable for the long-term planning of regional EMS infrastructure, strategic legislation as well as innovation focusing on the dispatch and operational routine for specific age-groups. Particularly the revealed urban–rural differences in today’s and tomorrow’s demand for EMS should be accounted for.

The fundamental challenge for further research will be to operationalize demography as one factor in a model of several factors for the total demand for EMS. We have found an ample variation of per capita demand in our study regions which could only partly be explained by demography. Thus it will be necessary to identify other significant factors, possibly depending on the individual study area, to evaluate their potential for future variation and to quantify this variation for demand projections. Especially in relation to factors rather intangible in numbers such as the inhibition threshold to call an ambulance, this task requires a multidisciplinary approach.
